# Ranking as a Procedure for Selecting a Replacement Variable in the Score Predicting the Survival of Patients Treated with Curative Intent for Colorectal Liver Metastases

**DOI:** 10.3390/medicina59112003

**Published:** 2023-11-15

**Authors:** Irena Plahuta, Matej Mencinger, Iztok Peruš, Tomislav Magdalenić, Špela Turk, Aleks Brumec, Stojan Potrč, Arpad Ivanecz

**Affiliations:** 1 Clinical Department of Abdominal and General Surgery, University Medical Centre Maribor, Ljubljanska 5, 2000 Maribor, Slovenia; irena.plahuta@ukc-mb.si (I.P.); tomislav.magdalenic@gmail.com (T.M.); turk.spela98@gmail.com (Š.T.); aleks.brumec@student.um.si (A.B.); potrc13@gmail.com (S.P.); 2 Department of Surgery, Faculty of Medicine, University of Maribor, Taborska ulica 8, 2000 Maribor, Slovenia; 3 Faculty of Civil Engineering, Transportation Engineering, and Architecture, University of Maribor, Smetanova ulica 17, 2000 Maribor, Slovenia; matej.mencinger@um.si (M.M.); iztok.perus@um.si (I.P.); 4 Institute of Mathematics, Physics and Mechanics, Jadranska 19, 1000 Ljubljana, Slovenia; 5 Faculty of Natural Science and Engineering, University of Ljubljana, Aškerčeva cesta 12, 1000 Ljubljana, Slovenia

**Keywords:** colorectal cancer, liver metastases, inflammation, ranking, survival

## Abstract

*Background and Objectives:* The issue of a missing variable precludes the external validation of many prognostic models. For example, the Liverpool score predicts the survival of patients undergoing surgical therapy for colorectal liver metastases, but it includes the neutrophil–lymphocyte ratio, which cannot be measured retrospectively. *Materials and Methods:* We aimed to find the most appropriate replacement for the neutrophil–lymphocyte ratio. Survival analysis was performed on data representing 632 liver resections for colorectal liver metastases from 2000 to 2020. Variables associated with the Liverpool score, C-reactive protein, albumins, and fibrinogen were ranked. The rankings were performed in four ways: The first two were based on the Kaplan-Meier method (log-rank statistics and the definite integral $I_{S}$ between two survival curves). The next method of ranking was based on univariate and multivariate Cox regression analyses. *Results:* The ranks were as follows: the radicality of liver resection (rank 1), lymph node infiltration of primary colorectal cancer (rank 2), elevated C-reactive protein (rank 3), the American Society of Anesthesiologists Classification grade (rank 4), the right-sidedness of primary colorectal cancer (rank 5), the multiplicity of colorectal liver metastases (rank 6), the size of colorectal liver metastases (rank 7), albumins (rank 8), and fibrinogen (rank 9). *Conclusions:* The ranking methodologies resulted in almost the same ranking order of the variables. Elevated C-reactive protein was ranked highly and can be considered a relevant replacement for the neutrophil–lymphocyte ratio in the Liverpool score. These methods are suitable for ranking variables in similar models for medical research.

## 1. Introduction

Colorectal cancer is the third-most-frequent malignant disease, with 1,800,000 people affected worldwide [1]. At diagnosis, the disease is disseminated in 15% to 25% of patients, and the liver is the most frequent site of distant metastases [2]. Another 25% of patients develop metachronous metastases [2]. The 5-year overall survival rate after the radical resection of colorectal liver metastases in well-selected patients is 47–60%. However, the disease recurs in 40–75% of patients, and the liver is involved in half of such patients [2].

Traditionally, factors associated with prognosis in patients with colorectal liver metastases have been associated with the characteristics of the primary tumor or metastases, including somatic mutations [3,4]. As a result, more than a dozen prognostic scoring systems have been developed, and some have been externally validated [5]. Recently, the paradigm of prognostication shifted toward the host’s immune system [6]. Furthermore, an inflammatory response can be identified by the ratios between blood cells or the proteinaceous inflammatory markers of the acute phase response [7,8,9,10]. These could be C-reactive proteins, albumins, or fibrinogens [11,12].

In 2019, Dupre et al. [13] developed and validated the Liverpool score. This is a prognostic scoring system that includes the systemic inflammatory response, expressed as the neutrophil–lymphocyte ratio, to predict the survival of patients undergoing surgical therapy for colorectal liver metastases [13]. The factors used in prognostic scoring systems are usually part of a routine work-up. Missing variables that cannot be obtained retrospectively represent a challenging issue for validation [14,15].

The aim of the study was to rank original variables and proteinaceous inflammatory markers as candidates that could replace the neutrophil–lymphocyte ratio, further validating the prognostic scoring system for patients treated with curative intent for colorectal liver metastases. To the best of our knowledge, it is the only prognostic factor ranking that is based on the definite integral $I_{S}$ calculation.

## 2. Materials and Methods

### 2.1. Materials

#### 2.1.1. Patients

A retrospective review of a prospectively maintained database of 632 patients who underwent surgical treatment for colorectal liver metastases at a specialized referral center for hepato-pancreato-biliary surgery (i.e., the Clinical Department of Abdominal and General Surgery of University Medical Centre Maribor in Slovenia) was performed. The study period lasted from 1 January 2000 to 31 December 2020.

This study was based on the intention-to-treat principle. Routinely available variables were obtained from the database and analyzed, as the patients were subjected to routine diagnostics. The diagnostic included a colonoscopy, blood work, and medical imaging, and is described in [16,17]. Patients were considered by a multidisciplinary team, and those with metastases confined to the liver were considered for liver resection, radiofrequency ablation, or a combination of the two [2]. Liver resections were defined as either anatomically minor or major, with a major liver resection involving three or more adjacent liver segments [18]. The principle of being “radical but conservative” (radical in oncological terms and conservative in preserving nontumoral liver parenchyma) was employed [19].

The routine blood work did not include determining the white blood cell count, but included the assessment of proteinaceous inflammatory markers: C-reactive protein, albumins, and fibrinogen [12]. They were measured by automatized analyzers from the peripheral vein blood of patients one day before the scheduled liver procedure [20]. Fibrinogen was obtained in a standard coagulation panel. A postoperative histopathological examination confirmed the diagnosis of colorectal liver metastases.

Overall survival was defined and calculated as the period from the first therapy for colorectal liver metastases (an upfront liver resection or neoadjuvant chemotherapy before liver resection) to the last follow-up visit or death.

Patients consented prior to the surgery to their anonymous data being used for research. Therefore, their records were anonymized and deidentified before analysis. Ethical approval for this study was obtained from the National Medical Ethics Committee of the Republic of Slovenia (0120-455/2020/3, 29 October 2020). All methods were used in accordance with the relevant guidelines and regulations.

#### 2.1.2. The Liverpool Score

The Liverpool score, introduced by Dupre et al., was analyzed [13]. It contains the following parameters:

1. The American Society of Anesthesiologists Classification (ASA classification) [21];
2. The location of primary colorectal cancer in the right colon (cecum, ascending colon, hepatic flexure, and proximal two-thirds of the transverse colon) or the left colon and rectum;
3. The malignant infiltration of primary colorectal cancer lymph nodes;
4. Size of the largest colorectal liver metastasis;
5. The multiplicity of colorectal liver metastases;
6. A positive liver resection margin (less than 1 mm between tumor cells and the transection plain under a histopathologic examination) [22]—a negative resection margin was defined as a clear microscopic margin [2];
7. The neutrophil–lymphocyte ratio.

#### 2.1.3. Proteinaceous Inflammatory Markers

The neutrophil–lymphocyte ratios could not be obtained retrospectively because the white blood cell count was not a part of our routine preoperative blood tests. 

The idea was to substitute this factor with a proteinaceous inflammatory marker, since both reflect the systemic inflammatory response. Therefore, three routinely measured candidate variables were proposed.

C-reactive protein (mg/L) is a positive acute-phase protein. Its elevated value has previously been shown to predict worse survival [9,23]. Values of ≥6 mg/L are considered elevated by the accredited institutional laboratory.

Albumin (g/L) is a negative acute-phase protein, and its low value is a predictor of poor overall survival [24]. The accredited institutional laboratory considers values between 32 and 55 g/L as normal.

Fibrinogen (g/L) is a positive acute-phase protein. A low value is observed in patients with reduced synthesis due to hepatic impairment, consumption, or hemodilution [25]. Its elevated value has been shown to predict worse survival [26]. According to the accredited institutional laboratory, values of <2.19 g/L are low, those from 2.2 to 4.2 g/L are normal, and those of ≥4.2 g/L are elevated.

#### 2.1.4. Inclusion and Exclusion Criteria

The inclusion criterion included patients who underwent their first liver resection for colorectal liver metastases between 2000 and 2020. The exclusion criteria were radiofrequency ablations or their combinations with liver resections, as well as 90-day postoperative mortality.

### 2.2. Methods

#### 2.2.1. Statistical Analysis

IBM SPSS for Windows Version 28.0 (IBM Corp., Armonk, NY, USA) and Microsoft Excel 2021 (Microsoft Corp., Redmond, WA, USA) were used for statistical computations. Categorical variables are displayed as frequencies with percentages. Continuous variables are displayed as medians (minimum–maximum; interquartile range). Survival is displayed in months (95% confidence interval). A *p* value of <0.05 was considered statistically significant.

Categorical variables were transformed binarily, as in the original prognostic scoring system [13]. The ASA classification [21] was divided into ASA 1 vs. ASA 2 or 3 to equilibrate the number of group members. 

To convert continuous variables into binary categorical variables, our approach was as follows: An elevated C-reactive protein was defined as 5 mg/L, as this was the highest value in its referential range [11]. A median value was used for the size of the metastasis because it had no referential range. Albumins had a referential range from 32 to 55 g/L, but the median value was used to ensure statistical differences among the groups. Fibrinogen exhibited statistical insignificance irrespective of the chosen (threshold) cut-off value. 

In our survival analysis, two functions dependent on time were of detailed interest. The first was the survival function S(t), defined as the probability of surviving at least until time t. The hazard function h(t) indicated the conditional probability of dying at time t, having survived until that time [27]. 

#### 2.2.2. Ranking Method #1

The survival curve is the graph of S(t) against *t*. The Kaplan-Meier method was used to estimate the survival curve from the observed survival times without assuming an underlying probability distribution [27]. A comparison of survival curves (life tables) was made using the log-rank test with chi-square statistics [27].

#### 2.2.3. Ranking Method #2

Based on the Kaplan-Meier analysis, the second ranking was performed using the integral [5] $I_{S}=\int_{t=0}^{t_{max}}\left[M\left(t\right)-m\left(t\right)\right]dt\;$, which was calculated in Microsoft Excel.

In most cases, the compared groups were formed according to the median value of the variable. The curve of a group with a more favorable prognostic factor (i.e., the group above the median) was denoted as S=M(t), while the curve of a group below the median was denoted as S=m(t).

The survival functions were step functions; therefore, integral $I_{S}$ was obtained as a sum of the differences between curves. A potential distance between curves would result in an increase in integral $I_{S}.$ Meanwhile, a variable with overlapping curves $S=M\left(t\right)$ and $S=m\left(t\right)$ would decrease integral $I_{S}$ and be ranked lower than a variable without overlapping curves, even though the two curves define the same area [23].

This integral on the interval from t=0 to $t_{max}$, expressed in months, can be assumed to be linearly proportional to the rank of a certain variable, and can be considered a quantitative measure of the sensitivity and impact of a certain variable relative to the scoring system [28].

#### 2.2.4. Ranking Method #3

Cox’s proportional hazard model is analogous to multiple regression models. It enables the testing of the difference between the survival times of particular groups of patients while allowing for other factors [27]. The response (dependent) variable is a hazard. The hazard is the probability of dying having survived up to a given point in time, or the risk of death at that moment. The hazard ratio (Exp(b)) does not depend on time. For example, if Exp(b) were 1.15, it would indicate that a person from a group with an unfavorable prognostic factor would be 15% more likely to die at any time than a patient from a group with a favorable prognostic factor [27].

Therefore, the third ranking was performed via univariate Cox regression analysis, and the two groups were compared. The same variables were ranked according to the hazard ratio $\left(Exp\left(b\right)\right)$ and *p* values. Assuming that a variable with a higher Exp(b) factor would have a higher impact on survival than a variable with a lower $Exp\left(b\right)$ factor was justified.

#### 2.2.5. Ranking Method #4

Finally, the fourth ranking was performed via multivariate Cox regression analysis, directly comparing the variables considered in the model. All computations were carried out via the SPSS program using the “Enter” method. This meant that the variables were manually proposed for the computation, and the system later added or removed none of them. The variables were ranked as mentioned above.

The final ranking was based on integral $I_{S}$ and multivariate Cox regression analysis. Integral $I_{S}$ is a quantitative measurement of the variable’s impact on survival and resolves the issue of overlapping curves. The multivariate Cox regression analysis revealed the behavior of the variable in the multivariate system. 

## 3. Results

### 3.1. Study Population

From 1 January 2000 to 31 December 2020, 632 procedures for colorectal liver metastases were performed. Their detailed descriptions have already been published elsewhere [16]. For this study, a cohort of 371 patients who had undergone an initial liver resection was selected. The follow-up period was concluded on 31 December 2022, with a median follow-up of 139 (120.5–157.5) months. The participants included 243 (65.5%) males and 128 (34.5%) females. Their median age was 65 (27–85; 15) years. Synchronous metastases were present in 187 (50.4%) patients, and metachronous metastases were present in 184 (49.6%) patients. In addition, 168 (45.3%) patients received neoadjuvant chemotherapy. The liver parenchyma was normal in 238 (64.1%) patients, liver steatosis was present in 130 patients (35.1%), and liver cirrhosis was present in 3 patients (0.8%). There were 263 (70.9%) minor and 108 (29.1%) major liver resections. The median overall survival was 40 months (36.4–43.6). 

### 3.2. Ranking of Survival Factors

**Figure 1:**
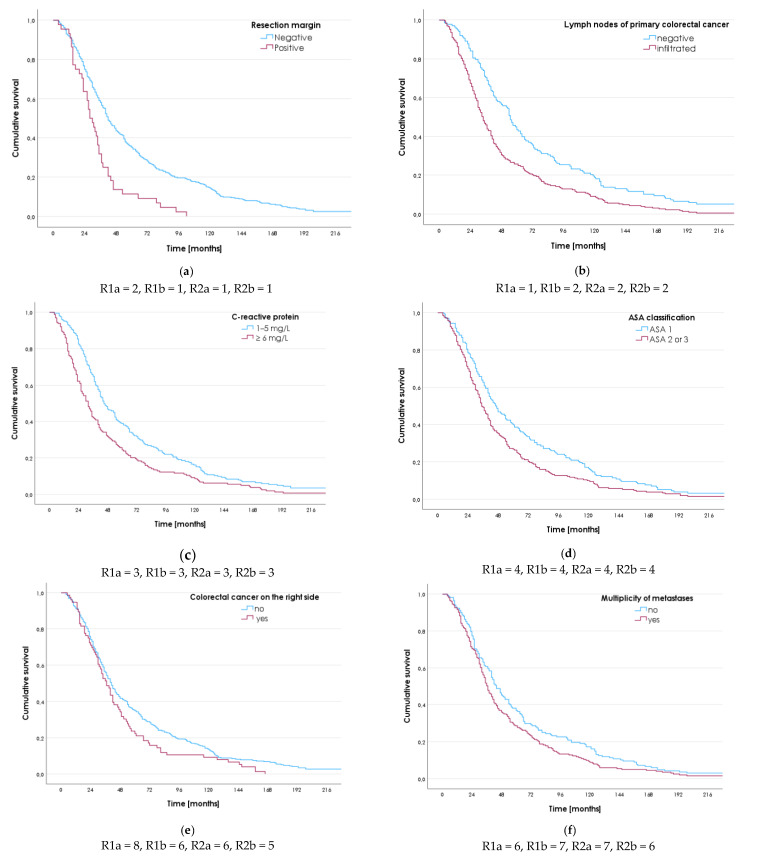

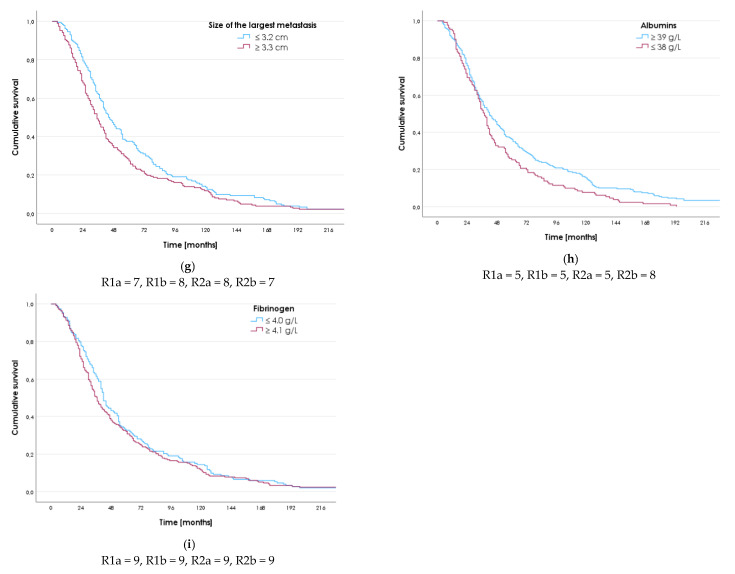
Summary of the ranking of survival factors. (**a**) The resection margin state, (**b**) Infiltration of lymph nodes of primary colorectal cancer, (**c**) C-reactive protein, (**d**) ASA classification, (**e**) Colorectal cancer on the right side, (**f**) Multiplicity of liver metastases, (**g**) Size of the largest liver metastasis, (**h**) Albumins, (**i**) Fibrinogen. R1a—ranking via the Kaplan-Meier method; R1b—ranking via integral $I_{S}$; R2a—ranking via univariate Cox analysis; R2b—ranking via multivariate Cox analysis. ASA: American Society of Anesthesiologists.

Nine variables were analyzed via the Kaplan-Meier method (Table 1a), the integral $I_{S}\;$between the Kaplan-Meier curves (Table 1b), univariate Cox regression analysis (Table 2a), and multivariate Cox regression analysis (Table 2b). A summary is given in Figure 1.

## 4. Discussion

The most important finding of our research was that the C-reactive protein was ranked relatively high by all four methods and, therefore, seems to be a promising factor for further analysis.

The first prognostic scoring system for colorectal liver metastases was published in 1996 [29]. Since then, more than two dozen prognostic scoring systems have been developed [5,14]. However, tools must be validated before they are applied to clinical practice [14,30,31,32,33]. A retrospectively unobtainable value or variable usually precludes external validation [15].

In 2019, Dupre et al. developed and validated the Liverpool score [13], is a prognostic scoring system that includes the systemic inflammatory response expressed as the neutrophil–lymphocyte ratio [13]. Under the influence of cytokines, neutrophils can exert carcinogenic and anticarcinogenic effects in the tumor microenvironment. Meanwhile, relative lymphocytopenia may reflect a poorer cell-mediated immune response relative to cancer. The immune microenvironment influences the neutrophil–lymphocyte ratio. The ratio reflects the level of the host’s immune surveillance obstacle [34]. Its prognostic role in surgical patients with colorectal liver metastases has previously been shown [35].

However, the white blood cell count was not a part of our routine preoperative blood tests; hence, the neutrophil–lymphocyte ratio could not be obtained retrospectively. Therefore, three routinely measured candidate variables were proposed in order to find the most suitable replacement for the neutrophil–lymphocyte ratio: C-reactive protein, albumins, and fibrinogen. Their prognostic value in cancer survival has already been well established [9,26,36].

Our comprehensive analysis of the survival factors of 371 patients undergoing surgical treatment for colorectal liver metastases utilized multiple ranking methods, including Kaplan–Meier analysis, the integral $I_{S}$ calculation, and Cox regression analyses. Survival factors were ranked according to their association with patient survival. 

Our decision to employ multiple ranking methods was driven by the intention to provide a robust and comprehensive assessment of prognostic factors. Each method introduced a unique perspective to the analysis. While Kaplan–Meier analyses offered a visual representation of survival curves, the integral $I_{S}$ calculation allowed for a quantitative measurement of the sensitivity and the impact of each variable on the scoring system [28]. Univariate and multivariate Cox regression analyses further elucidated the individual and collective contributions of these factors.

Indisputably, the oncologically non-radical resection of colorectal liver metastases (positive resection margin) and infiltrated lymph nodes of colorectal cancer received the highest ranks among the variables. The rates of non-radical resection of colorectal liver metastases were 10–30% in the most experienced centers, with a rate of 11.9% in [22]. The worse overall survival risk observed in the positive resection margin group was approximately 80% higher than that in the negative resection margin group (Table 2b), with an integral $I_{S}\;$of 28.1 months.

Our patients underwent surgery for disseminated cancer disease. Additionally, 62.8% of patients exhibited infiltrated lymph nodes with primary tumors. The risk of worse overall survival in the group with infiltrated lymph nodes was approximately 55% higher than that in the group with non-infiltrated lymph nodes (Table 2b), with an integral $I_{S}\;$of 26.6 months. The infiltration of lymph nodes is used in several prognostic scoring systems, including the most well-known Memorial Sloan Kettering Cancer Centre Clinical Risk Score used by Fong et al. [29,37,38]. 

The C-reactive protein emerged as a consistently high-ranking factor across all methods, indicating its significant association with patient survival, as already reported in patients with colorectal liver metastases [9,23,36,39]. It ranked third place. The elevated C-reactive protein value group had an approximately 35% higher risk of worse overall survival compared to the normal C-reactive protein value group (Table 2b), with an integral at $I_{S}$ = 20.6 months. This suggests that the C-reactive protein holds promise as a surrogate for the neutrophil–lymphocyte ratio in prognostic scoring systems, offering a practical alternative for cases where neutrophil–lymphocyte ratio measurements are not feasible. A similar method was proposed by Malik et al., who assembled a prognostic scoring system in 2007 [40]. The risk factors were the number of liver metastases being ≥8 and inflammatory response in the form of a neutrophil–lymphocyte ratio of >5:1 or a C-reactive protein level of >10 mg/L [40]. This prognostic scoring system was recently validated for oligometastatic colorectal cancer [41]. 

Moreover, along with its role in inflammation and cancer prognosis, elevated serum C-reactive protein values have been associated with the risk of malnutrition and neoplastic cachexia [34]. This protein can also be elevated in infectious and auto-immune diseases [20,42].

ASA classifications 2 and 3 [21] were stable at the fourth rank. The risk of worse overall survival was approximately 33% higher in the ASA 2 and 3 groups than in the ASA 1 group (Table 2b), and the integral $I_{S}$ was 16.6 months. A worse survival rate in the ASA 2 or 3 group was proven with regard to the outcomes of radical nephroureterectomy [43]. To our knowledge, Dupre et al. [13] were the first to utilize the ASA classification in a prognostic scoring system for colorectal liver metastases.

The ranking of albumin, a primary tumor on the colon’s right side, and sizes and multiplicity of the colorectal liver metastases varied.

In this cohort, 20.4% of the patients had primary colorectal cancer in the right colon. In the study conducted by McCracken et al., 37% of primary colorectal cancer cases occurred in the right colon [44]. The prognosis of right-sided colorectal cancer is worse [44,45]. In our study, the risk of the right-sided group was approximately 24% higher than that of the non-right-sided group (Table 2b), with an integral $I_{S}$ = 13.3 months. It was ranked fifth.

Furthermore, we believed that the greater the number of lesions and the larger their size, the worse the prognosis of colorectal liver metastases would be. However, whether or not the resection of colorectal liver metastases was able to achieve negative resection margins, survival was the same regardless of the number of lesions [2]. Our analysis ranked the multiplicity of the metastases group at sixth place. The risk of worse overall survival in the multiple metastases group was approximately 21% higher than in the solitary metastasis group (Table 2b), with a statistical significance close to <0.05, and the integral $I_{S}$ was 12.1 months [46]. The size of the largest colorectal liver metastasis was ≥3.3 cm, and it was ranked seventh, with a risk approximately 14% higher than that of the smaller colorectal liver metastases group (Table 2b). The difference was statistically insignificant (*p* = 0.217), but the integral between groups was still $I_{S}$ = 11.1 months.

The most interesting ranking was observed for the low-albumin group. A low level of albumins is important in colorectal cancer survival, and is used in prognostic scoring systems [24,47]. The survival factor was given the fifth rank according to the Kaplan-Meier, integral $I_{S}$, and univariate Cox regression analyses, with a risk of worse overall survival approximately 33% higher than the normal-albumin-level group (Table 2a). However, with respect to multivariate Cox regression analysis, it was ranked eighth, with a risk approximately 14% higher than that in the normal-albumin-level group (Table 2b) and an insignificant *p* value = 0.270, but the integral between groups was $I_{S}$ = 14.7 months.

Hyperfibrinogenemia was proven to be a prognostic sign of worse overall survival in colorectal cancer by Li et al. and Tang et al. [26,48,49]. However, it was not a risk factor in multivariate analysis, as reported by Yamashita et al. and Pedrazzani et al. [50,51]. The high-fibrinogen group unanimously took ninth place, with no statistical significance (*p* value = 0.872) (Table 2b). From a medical point of view, the issue with this candidate proteinaceous inflammatory marker was the narrow range of its reference value, at 2.2–4.2 g/L. 

Among other proteins, the carcinoembryonic antigen was measured before liver resection for colorectal liver metastasis as a part of routine work-up and after resection as a part of the standard surveillance protocol [52]. Its elevation from the normal range was a signal indicating that the patient should be checked for disease recurrence [52]. However, the carcinoembryonic antigen had moderate sensitivity with respect to recurrence detection, as well as relatively high rates of false positivity [53].

Recently, circulating tumor deoxyribonucleic acid (ctDNA) has emerged as a potential biomarker, especially for predicting recurrence in patients with colorectal liver metastasis [53]. Patients with detectable ctDNA after liver resection had a higher chance of recurrence and shorter durations of overall survival compared to patients without detectable ctDNA. On the contrary, there was no association between detectable ctDNA before surgery and survival [53].

To summarize, our study builds upon the foundation laid by previous research, particularly Dupre et al., Malik et al., and Frühling et al. [13,20,40,54]. By ranking variables and proposing the C-reactive protein as a potential substitute, we aimed to extend the utility of prognostic scoring systems and to address the challenges posed by unobtainable retrospective values, such as the neutrophil–lymphocyte ratio.

In essence, the combination of these ranking methodologies adds depth to our analysis, offering a comprehensive view of the variables’ association with patient survival. The consistency in the ranking order across methods, as highlighted in our results, strengthens the reliability of our findings and underscores the potential significance of C-reactive proteins as a substitute for the neutrophil–lymphocyte ratio. 

Future directions for this research include the development of an integrative computational and experimental model that employs neural networks. This approach, which will go beyond this initial statistical framework, is designed to provide a more comprehensive and applicable model for the medical community. 

It is crucial to acknowledge the limitations of our retrospective study, including sample size constraints. Forthcoming research endeavors could benefit from larger cohorts and prospective designs. Additionally, we recognize the need for external validation of our findings in diverse patient populations.

## 5. Conclusions

Our study offers a nuanced understanding of prognostic factors in surgical patients with colorectal liver metastasis. The integration of various ranking methods enhances the robustness of our findings and underscores the potential clinical relevance of our proposed substitute for the neutrophil–lymphocyte ratio in prognostic models. The most important finding is that the C-reactive protein was ranked relatively high by all four methods, and this introduces it as a compelling candidate for further investigation. These methods are suitable for ranking variables in similar models that are used in medical research.

## Figures and Tables

**Table 1:** Ranking of survival factors via Kaplan-Meier analysis (a) and the integral $I_{S}$ of the cumulative functions of the two selected groups that differed in terms of overall survival (b).

		(a) Kaplan-Meier Analysis			(b) Integral	
Variable	*N* (%)	Chi-Square	*p* Value	Rank	$I_{S}$ (Months)	Rank
Infiltrated lymph nodes of a primary tumor	233 (62.8%)	21.6	<0.001	1	26.6	2
Positive resection margin	44 (11.9%)	20.0	<0.001	2	28.1	1
C-reactive protein ≥ 6 mg/L	164 (44.2%)	16.5	<0.001	3	20.6	3
ASA classification 2 or 3	213 (57.4%)	8.4	0.004	4	16.6	4
Albumins ≤ 38 g/L	131 (35.3%)	6.7	0.010	5	14.7	5
The multiplicity of liver metastasis	203 (54.7%)	5.1	0.024	6	12.3	7
The largest liver metastasis ≥ 3.30 cm	187 (50.4%)	4.8	0.028	7	11.1	8
Primary tumors on the right side	76 (20.4%)	4.2	0.040	8	13.3	6
Fibrinogen ≥ 4.1 g/L	218 (58.7%)	0.5	0.467	9	3.2	9

ASA: American Society of Anesthesiologists.

**Table 2:** Ranking of survival factors by univariate (a) and multivariate Cox regression analyses (b).

	(a) The Univariate Cox Analysis					(b) The Multivariate Cox Analysis				
	*p* Value	$\mathrm{Hazard}\;\mathrm{Ratio}\;(Exp\left(b\right))$	$95.0\%\;\mathrm{CI}\;\mathrm{for}\;Exp\left(b\right)$		Rank	*p* Value	$\mathrm{Hazard}\;\mathrm{Ratio}\;(Exp\left(b\right))$	$95.0\%\;\mathrm{CI}\;\mathrm{for}\;Exp\left(b\right)$		Rank
			Lower	Upper				Lower	Upper	
Positive resection margin	<0.001	2.04	1.5	2.8	1	<0.001	1.79	1.3	2.5	1
Infiltrated lymph nodes of a primary tumor	<0.001	1.65	1.3	2.0	2	<0.001	1.55	1.2	1.9	2
C-reactive protein ≥ 6 mg/L	<0.001	1.53	1.2	1.9	3	0.012	1.35	1.1	1.7	3
ASA classification 2 or 3	0.004	1.35	1.1	1.7	4	0.009	1.33	1.1	1.6	4
Albumins ≤ 38 g/L	0.011	1.33	1.1	1.6	5	0.270	1.14	0.9	1.4	8
Primary tumor on the right side	0.042	1.30	1.0	1.7	6	0.137	1.24	0.9	1.6	5
The multiplicity of liver metastasis	0.026	1.26	1.0	1.6	7	0.087	1.21	1.0	1.5	6
The largest liver metastasis ≥ 3.30 cm	0.030	1.25	1.0	1.5	8	0.217	1.14	0.9	1.4	7
Fibrinogen ≥ 4.1 g/L	0.471	1.080	0.9	1.3	9	0.872	0.982	0.8	1.2	9

ASA: American Society of Anesthesiologists; CI: confidence interval.

## Data Availability

The data presented in this study are available upon request from the corresponding author. The data are not publicly available, because they are confidential patient data.
